# Depression and its associated factors among HIV/AIDS patients attending ART clinics at Gimbi General hospital, West Ethiopia, 2018

**DOI:** 10.1186/s13104-019-4553-0

**Published:** 2019-08-20

**Authors:** Muktar Abadiga

**Affiliations:** School of Nursing and Midwifery, Institute of Health Sciences, Wollega University, Nekemte, Ethiopia

**Keywords:** Depression, HIV/AIDS patients, Gimbi General hospital

## Abstract

**Objective:**

The aim of this study was to assess the prevalence and factors associated with depression among people living with HIV/AIDS attending Gimbi General hospital, West Ethiopia. Institutional based cross-sectional study was conducted on 404 HIV/AIDS patients, from March 01 to March 30, 2018. Multivariable logistic regression was used to determine factors associated with depression. Possible association and statistical significance were measured using odds ratio at 95% confidence interval and P-value less than 0.05.

**Results:**

A total of 393 HIV/AIDS patients were included in this study. Out of this, 41.7% had depression. Perceived social stigma (AOR = 6.98, 95% CI 3.07, 15.86), opportunistic infection (AOR = 9.38, 95% CI 4.21, 20.89), adverse drug reaction (AOR = 3.73, 95% CI 1.58, 8.81), absence of family/social support (AOR = 9.97, 95% CI 3.57, 27.86), and presence of other chronic diseases (AOR = 6.14, 95% CI 1.66, 22.68) were significantly associated with depression. The level of depression among HIV/AIDS patient in this study was high. The clinician should early recognize and treat drug side effects, early detect and manage opportunistic infection and other chronic diseases, and give health information about the disease for the community to reduce social stigma.

## Introduction

Depression is a common mental disorder presents with depressed mood, loss of interest, feelings of guilt, disturbed sleep, and poor concentration [1]. It is the leading cause of disability and the fourth leading contributor to the global burden of disease [2, 3]. About 350 million people were affected worldwide, and the life-time risk of depression is one in five women and one in ten men [4, 5]. In Ethiopia, the pooled prevalence of depression is 11% [6]. Depression is one of the most common mental disorders people with HIV/AIDS experience [7, 8]. The prevalence of depression on HIV-infected patients in Brazil is 32% [9]. Other studies conducted in different countries on prevalence of depression among HIV patients showed 26.7% in Cameroon [10], 18.9% in Malawi [11] and 40.9% in China [12]. In Ethiopia, the study done in different part of a country showed 48.6% in Hawassa [13], 14.6% in Aksum town [14], 45.8% in Harar town [15], and 35.5% in Addis Ababa [16]. The level of depression is related to the severity of symptoms of HIV infection, and the presence of HIV/AIDS increases the risk of mental disorders [17–19].

Depression and HIV/AIDS are estimated to be the world’s two leading causes of disability by 2030 [20]. Depression among PLHIV influences not only the health status of PLHIV but also has a negative effect on ART adherence [21]. Depression affects the ability to comply with HIV/AIDS treatment, as well as quality of life and lifespan [22, 23]. Depression also has destructive effects on the self-care behaviors necessary for management of HIV [24]. Studies showed that factors such as unemployment, age, CD4 count, sex, educational status, drug side effects, family/social support, stigma, stage of HIV, living companion, marital status, monthly income and opportunistic infection were predisposed HIV/AIDS patients to depression [10–16].

Although the prevalence of depression is high among HIV/AIDS patients, few studies have been conducted in Ethiopia and no study has been conducted at Gimbi General hospital. This study was done to fill this research gap which might provide evidence for the future’s effective prevention and treatment of depression in people living with HIV/AIDS. Therefore; this study was aimed to assess the prevalence and associated factors of depression among people living with HIV/AIDS attending Gimbi General hospital, West Ethiopia.

## Main text

### Methods

#### Study setting and population

This study was conducted at Gimbi General hospital from March 01 to March 30, 2018. The institutional based cross-sectional study design was employed. All ART patients on treatment follow up at Gimbi General hospital was the source population and the sampled ART patients who had treatment follow up during the study period was the study population. Patients whose ages are 18 years and above were included in the study. Patients who are eligible but not willing to take part in the study were excluded.

#### Sample size determination and sampling techniques

The sample size was calculated using the formula for estimation of a single population proportion. A 48.6% (0.486) proportion of HIV/AIDS patients who had depression was taken from similar a study done in Hawassa University Comprehensive Specialized Hospital [13], and by adding a non-response rate of 5%, a total of 404 ART patients were enrolled in the study. Simple random sampling method was used to select the study participants.

#### Data collection tool and procedures

The data collection tool includes socio-demographic characteristics, clinical and behavioral variables, nine-item Patient Health Questionnaire (PHQ-9), The Eight-Item Morisky Medication Adherence Scale, Oslo 3-item social support scale and an 11-item HIV stigma scale. Depression was defined by a PHQ-9 score ≥ 5 [25]. Adherence was defined as adherent with a Morisky Medication Adherence Scale score of 0 and non-adherent with a score of ≥ 1. Oslo 3-item social support scale has three categories: “poor support” 3–8, “moderate support” 9–11 and “strong support” 12–14 [26]. Study participants were classified as having or not having stigma using the mean of the stigma variable as cutoff point [27, 28].

#### Data processing and analysis

The data were coded, checked, cleaned and entered into Epi data version 3.1 and then exported into SPSS window version 20.0 for analysis. Bivariate analysis was done to find association between each independent variable with depression. Finally, multivariable logistic regression was used to find out the independent variables which best predict depression. Possible association and statistical significance were measured using odds ratio at 95% confidence interval and P-value less than 0.05.

#### Data quality control

The questionnaire was translated into the local language and then translated back to English by expertise for consistency. Five percent (5%) of the questionnaire was pre-tested on ART patients having treatment follow up at Nekemte referral hospital. One-day training was also given for data collectors and supervisors.

### Results

#### Socio-demographic characteristics of participants

Out of the total of 404 study participants sampled, 393 were participated; making a response rate of 97.3%. From the total of 393 participants, 177 (45%) were male, and 216 (55%) were females. One hundred forty-two, (36.1%) lie in the age group between 18 and 28 years, and the mean age was 25.6 years with ± 9.45 SD. Regarding religion, 182 (46.3%) were Protestants. Concerning marital status, 174 (44.3%) were single followed by married, 132 (33.6%). Concerning education, 168 (42.7%) were completed grade 9–12, and 128 (32.6%) were completed grade 1–8. Regarding income, 108 (27.5%) gets monthly income < 500 Ethiopian birrs followed by 500–1000 Ethiopian birr which accounts 98 (24.9%) (Table 1).

**Table 1:** Distribution of study participants by socio-demographic characteristics among HIV/AIDS patients at Gimbi General hospital, West Ethiopia, 2018 (n = 393)

Variables	Frequency	Percentage
Sex		
Male	177	45
Female	216	55
Total	393	100
Ethnicity		
Oromo	234	59.5
Amhara	106	27.0
Tigre	24	6.1
Gurage	16	4.1
Others	13	3.3
Total	393	100
Age		
18–28	142	36.1
29–38	140	35.6
39–48	56	14.2
≥ 48	55	14.0
Total	393	100
Religion		
Protestant	182	46.3
Orthodox	106	27.0
Muslim	49	12.5
Catholic	46	11.7
Others	10	2.5
Total	393	100
Marital status		
Married	132	33.6
Single	174	44.3
Divorced	49	12.5
Widowed	38	9.7
Total	393	100
Educational status		
No formal education	30	7.6
Primary school (1–8)	128	32.6
Secondary (9–10)	168	42.7
Degree and above	67	17.0
Total	393	100
Occupational status		
Gov’nt employee	52	13.2
Private employee	121	30.8
Farmer	95	24.2
Merchant	109	27.7
Others	16	4.1
Total	393	100
Monthly income		
< 500	108	27.5
500–1000	98	24.9
1001–1500	78	19.8
1501–2000	75	19.1
> 200	34	8.7
Total	393	100
Living companion		
Yes	264	67.2
No	129	32.8
Total	393	100

#### Clinical and behavioral characteristics of participants

One hundred eighty-one (46.1%) had CD4 count 200–500 cells and 133 (33.8%) had CD4 count 501–800 cells. Regarding the stage of HIV, 153 (38.9%) were on stage II and 117 (29.8%) were on stage I. One hundred seventy-seven (45%) had strong social support, 147 (37.4%) had moderate and the remaining 69 (17.6%) had poor social support. Majority of the study participants, 247 (62.8%) don’t experienced social stigma and 146 (37.2%) had experienced social stigma. Majority of the study participants, 363 (92.4%) had no history of other chronic illness and 270 (68.7%) had no current opportunistic infections. Majority of the study participants, 268 (68.2%) had no adverse drug reaction, and the remaining 125 (31.8%) had experienced an adverse drug reaction (Table 2).

**Table 2:** Distribution of study participants by clinical and behavioral factors among HIV/AIDS patients at Gimbi General hospital, West Ethiopia, 2018 (n = 393)

Variables	Frequency	Percentage
CD4 cell count		
< 200	63	16.0
200–500	181	46.1
501–800	133	33.8
> 800	16	4.1
Total	393	100
Stage of HIV		
Stage I	117	29.8
Stage II	153	38.9
Stage III	72	18.3
Stage IV	51	13.0
Total	393	100
Social support		
Poor	69	17.6
Moderate	147	37.4
Strong	177	45.0
Total	393	100
Perceived social stigma		
Yes	146	37.2
No	247	62.8
Total	393	100
Current substance use		
Yes	157	39.9
No	236	60.1
Total	393	100
Duration of disease		
< 1 year	54	13.7
1–5 years	137	34.9
5–10 years	148	37.7
> 10 years	54	13.7
Total	393	100
Adverse drug reaction		
Yes	125	31.8
No	268	68.2
Total	393	100
History of other chronic illness		
Yes	30	7.6
No	363	92.4
Total	393	100
History of OIs		
Yes	123	31.3
No	270	68.7
Total	393	100
Adherence to medication		
Yes	245	62.3
No	148	37.7
Total	393	100

#### Level of depression among the study participants

The level of depression among the study participants were measured using nine-item Patient Health Questionnaire (PHQ-9) which is a validated depression screening tool having score range from 0 to 27. Study participants who scored PHQ-9 score ≥ 5 were considered depressed and those who scored < 5 were considered not depressed. Accordingly, out of the total of 393 study participants, 229 (58.3%) had no depression and 164 (41.7%) had depression.

#### Bivariate logistic regression analysis

In bivariate analysis, socio-demographic characteristics such as monthly income, occupation and living companion showed significant association with depression at P-value less than 0.25. Clinical variables such as CD4 cell count, HIV stage, adherence to medication, adverse drug reaction, history of other chronic illness and opportunistic infection showed significant association. Behavioral variables such as social support, substance use and perceived social stigma also showed significant association with depression at P-value less than 0.25.

#### Multivariate logistic regression analysis

In the final model of logistic regression, social support, perceived social stigma, opportunistic infection, adverse drug reaction and presence of other chronic diseases were significantly associated with depression. Study participants who had poor social support were 9.97 times more likely to have depression than those who had strong social support (AOR = 9.97, 95% CI 3.57, 27.86). Study participants who had experienced social stigma were 6.98 times more likely to develop depression than those who had no experienced social stigma (AOR = 6.98, 95% CI 3.07, 15.86). Respondents who developed adverse drug reactions were 3.73 times more likely to have depression than those who don’t develop any adverse drug reaction (AOR = 3.73, 95% CI 1.58, 8.81). Study participants who had history of other chronic diseases were 6.14 more likely to develop depression than those who had no other chronic illness (AOR = 6.14, 95% CI 1.66, 22.68). Study participants who had history of opportunistic infection were 9.38 times more likely to have depression than those who had no history of opportunistic infection (AOR = 9.38, 95% CI 4.21, 20.89) (Table 3).

**Table 3:** Multivariate logistic regression analysis of factors associated with depression among HIV/AIDS patients at Gimbi General hospital, West Ethiopia, 2018 (n = 393)

Variables	Depression		COR (95%) CI	Unadjusted P value	AOR (95%) CI	Adjusted P value
	Yes (%)	No (%)				
Sex						
Male	72 (40.7%)	105 (59.3%)	0.924 (0.61, 1.38)	0.70	1.2 (0.8, 1.86)	0.35
Female	92 (42.6%)	124 (57.4%)	1		1	
Age (years)						
18–28	58 (40.8%)	84 (59.2%)	1		1	
29–38	57 (40.7%)	83 (59.3%)	0.99 (0.61, 1.59)	0.98	0.82 (0.28, 2.36)	0.72
39–48	22 (39.3)	34 (60.7%)	0.93 (0.49, 1.76)	0.84	0.37 (0.07, 1.96)	0.24
> 48	27 (49.1%)	28 (50.9%)	1.39 (0.74, 2.61)	0.29	0.82 (0.22, 3.07)	0.77
Religion						
Protestant	75 (41.2%)	107 (58.8%)	1		1	
Orthodox	44 (41.5%)	62 (58.5%)	1.01 (0.62, 1.64)	0.96	0.75 (0.27, 2.02)	0.57
Muslim	24 (49.0%)	25 (51.0%)	1.37 (0.72, 2.58)	0.33	0.63 (0.17, 2.28)	0.48
Catholic	16 (34.8%)	30 (65.2%)	0.76 (0.38, 1.49)	0.47	1.33 (0.32, 5.49)	0.69
Others	5 (50.0%)	5 (50.0%)	1.42 (0.39, 5.10)	0.58	2.10 (0.80, 8.22)	0.71
Marital status						
Married	51 (38.6%)	81 (61.4%)	1		1	
Single	73 (42.0%)	101 (58.0%)	1.14 (0.72, 1.82)	0.55	0.55 (0.20, 1.53)	0.25
Divorced	22 (44.9%)	27 (55.1%)	1.29 (0.66, 2.51)	0.44	0.95 (0.23, 3.89)	0.94
Widowed	18 (47.4%)	20 (52.6%)	1.42 (0.69, 2.95)	0.33	0.83 (0.13, 5.26)	0.84
Ethnicity						
Oromo	93 (39.7%)	141 (60.3%)	1		1	
Amhara	49 (46.2%)	57 (53.8%)	1.30 (0.82, 2.07)	0.26	2.18 (0.84, 5.61)	0.10
Tigre	11 (45.8%)	13 (54.2%)	1.28 (0.55, 2.98)	0.56	1.32 (0.28, 6.13)	0.71
Gurage	7 (43.8%)	9 (56.2%)	1.12 (0.42, 3.27)	0.75	1.69 (0.19, 14.6)	0.63
Others	4 (30.8%)	9 (69.2%)	0.67 (0.20, 2.25)	0.52	0.15 (0.01, 2.32)	0.17
Educational status						
No formal education	13 (43.3%)	17 (56.7%)	1.20 (0.50, 2.88)	0.67	1.08 (0.15, 7.50)	0.93
Primary school (1–8)	54 (42.2%)	74 (57.8%)	1.15 (0.62, 2.10)	0.64	2.05 (0.54, 7.67)	0.28
Secondary school (9–12)	71 (42.3%)	97 (57.7%)	1.15 (0.64, 2.05)	0.62	1.78 (0.55, 5.78)	0.33
College and above	26 (38.8%)	41 (61.2%)	1		1	
Residence						
Urban	55 (43.0%)	73 (57.7%)	1.07 (0.70, 1.65)	0.72	0.87 (0.34, 2.17)	0.76
Rural	109 (41.1%)	156 (58.9%)	1		1	
Occupation						
Government employee	19 (36.5%)	33 (63.5%)	1		1	
Private employee	50 (41.3%)	71 (58.7%)	1.22 (0.62, 2.39)	0.55	1.80 (0.48, 6.72)	0.37
Farmer	51 (53.7%)	44 (46.3%)	2.01 (1.00, 4.02)	0.04*	1.50 (0.38, 5.91)	0.55
Merchant	43 (39.4%)	66 (60.6%)	1.13 (0.57, 2.24)	0.72	2.06 (0.54, 7.77)	0.28
Others	1 (6.2%)	15 (93.8%)	0.11 (0.01, 0.94)	0.04*	0.07 (0.04, 1.31)	0.75
Income						
< 500 EB	51 (47.2%)	57 (52.8%)	0.62 (0.28, 1.36)	0.24*	0.92 (0.15, 5.54)	0.92
500–1000 EB	39 (39.8%)	59 (60.2%)	0.46 (0.20, 1.02)	0.05*	0.49 (0.07, 3.09)	0.44
1001–1500 EB	29 (37.2%)	49 (62.8%)	0.41 (0.18, 0.94)	0.03*	0.85 (0.13, 5.25)	0.86
1501–2000 EB	25 (33.3%)	50 (66.7%)	0.35 (0.15, 0.80)	0.01*	0.63 (0.10, 3.80)	0.62
> 2000 EB	20 (58.8%)	14 (41.2%)	1		1	
Living companion						
Yes	73 (27.7%)	191 (72.3%)	1		1	
No	91 (70.5%)	38 (29.5)	6.26 (3.93, 9.97)	0.01*	3.33 (1.58, 9.96)	0.09
CD4 cell						
< 200	31 (49.2%)	32 (50.8%)	0.44 (0.13, 1.41)	0.16*	0.49 (0.04, 5.70)	0.57
200–500	70 (38.7%)	111 (61.3%)	0.28 (0.096, 0.86)	0.02*	0.27 (0.02, 2.74)	0.26
501–800	52 (39.1%)	81 (60.9%)	0.29 (0.09, 0.88)	0.03*	0.31 (0.03, 3.14)	0.32
> 800	11 (68.8%)	5 (31.2%	1		1	
HIV stage						
Stage I	45 (38.5%)	72 (61.5%)	1		1	
Stage II	49 (32.0%)	104 (68.0%)	0.75 (0.45, 1.24)	0.27	0.74 (0.31, 1.76)	0.50
Stage III	33 (45.8%)	39 (54.2%)	1.35 (0.74, 2.45)	0.31	2.15 (0.74, 6.23)	0.15
Stage IV	37 (72.5%)	14 (27.5%)	4.22 (2.06, 8.67)	< 0.001*	2.31 (0.60, 8.86)	0.22
Family/social support						
Poor	45 (65.2%)	24 (34.8%)	2.79 (1.56, 4.99)	<0.001*	9.97 (3.57, 27.86)	0.000**
Moderate	48 (32.7%)	99 (67.3%)	0.72 (0.45, 1.14)	0.16*	1.38 (0.60, 3.13)	0.44
Strong	71 (40.1%)	106 (59.9%)	1		1	
Perceived stigma						
Yes	115 (78.8%)	31 (21.2%)	14.99 (9.04, 24.84)	< 0.001*	6.98 (3.07, 15.86)	0.000**
No	49 (19.8%)	198 (80.2%)	1		1	
Substance use						
Yes	128 (81.5%)	29 (18.5%)	24.52 (14.33, 41.95)	0.04*	5.06 (2.39, 10.75)	0.17
No	36 (15.3%)	200 (84.7%)	1		1	
Disease duration (years)						
< 1	20 (37.0%)	34 (63.0%)	1		1	
1–5	53 (38.7%)	84 (61.3%)	1.07 (0.56, 2.05)	0.83	0.64 (0.14, 2.78)	0.55
6–10	74 (50.0%)	74 (50.0%)	1.70 (0.89, 3.22)	0.10*	1.37 (0.32, 5.77)	0.66
> 10	17 (31.5%)	37 (68.5%)	0.78 (0.35, 1.73)	0.54	0.58 (0.10, 3.24)	0.54
Treatment duration (years)						
< 1	27 (37.5%)	45 (62.5%)	1		1	
1–5	65 (39.6%)	99 (60.4%)	1.09 (0.61, 1.93)	0.75	2.35 (0.77, 7.14)	0.13
6–10	68 (48.6%)	72 (51.4%)	1.57 (0.88, 2.81)	0.12*	4.0 (1.28, 12.51)	0.17
> 10	4 (23.5%)	13 (76.5%)	0.51 (0.15, 1.73)	0.28	3.21 (0.40, 25.69)	0.20
Adherence to medication						
Yes	59 (24.1%)	186 (75.9%)	1		1	
No	105 (70.9%)	43 (29.1%)	7.69 (4.85, 12.19)	0.003*	4.92 (1.84, 13.11)	0.061
Adverse drug reaction						
Yes	103 (82.4%)	22 (17.6%)	15.88 (9.24, 27.30)	< 0.001*	3.73 (1.58, 8.81)	0.003**
No	61 (22.8%)	207 (77.2%)	1		1	
History of other chronic illness						
Yes	20 (66.7%)	10 (33.3%)	3.04 (1.38, 6.68)	0.006*	6.14 (1.66, 22.68)	0.006**
No	144 (39.7%)	219 (60.3%)	1		1	
Opportunistic infection						
Yes	89 (72.4%)	34 (27.6%)	6.80 (4.22, 10.96)	< 0.001*	9.38 (4.21, 20.89)	0.000**
No	75 (27.8%)	195 (72.2%)	1		1	

**Significant at P-value < 0.05

### Discussion

The prevalence of depression among peoples living with HIV/AIDS in this study is 41.7%. This finding is consistent with study done in Harar (45.8%) [15], Tigray (43.9%) [18] and China (40.9%) [12]. However; it is lower than a study done in Fitche zonal hospital (76.7%) [29] and Hawassa (48.6%) [13]. On the other hand, it is higher than study done in Malawi (18.9%) [11], Addis Ababa (35.5%) [16], Aksum town (14.6%) [14] and Cameroon (26.7%) [10]. The difference might be due to variation in data collection tool, sample size and study participant’s variation. The prevalence of depression among people living with HIV/AIDS in this study is much higher than the general population in Ethiopia (11%) [6].

In this study, factors such as social support, perceived social stigma, opportunistic infection, adverse drug reaction and presence of other chronic diseases were significantly associated with depression. Study participant who have poor social support had more depression than those who have strong social support. This might be due to the fact that poor social support may leads to social isolation, which can be responsible for depression. This finding is consistent with study done in Addis Ababa [16], Hawassa [13] and China [12]. However; this finding is not consistent with study done in Cameroon [10], Harar [15], Aksum [14] and Malawi [11]. The finding of this study also showed that those participants who perceived social stigma had depression than who didn’t perceived social stigma. This might be due to chronic nature of the disease and the patient may prefer being alone to avoid stigma which may further lead to the development of depression. This result is similar with studies done in Malawi [11], Addis Ababa [16], Hawassa [13] and China [12].

Respondents who developed adverse drug reaction had more depressive symptoms than who didn’t develop any adverse drug reaction. This is due to the fact that ART side effects may disturb the normal functioning of HIV/AIDS patients, so that patients may feel hopeless and develop depressive symptoms. This finding is consistent with study done in Aksum town [14] and China [12]. The finding of this study also showed that those who had history of opportunistic infection were more likely to develop depression. This might be due to the fact that opportunistic infection may lead to dissatisfaction with one’s physical appearance, which might be a reason for the occurrence of depression. This finding is supported by study done in Addis Ababa [16] and Fitche zonal hospital [29].

In this study, respondents who had history of other chronic illness were more depressed than those who had no history of other chronic illness. This might be due to the occurrence of long-lasting stresses related to chronic diseases which may further leads to depressive symptoms. This finding is consistent with study conducted at Debrebirhan Referral Hospital [30]. Substance use, living companion, medication adherence and being female had no statistically significant association with depression in this study.

### Conclusion

The level of depression among HIV/AIDS patient in this study was high. Depression among people living with HIV/AIDS is underdiagnosed and further large-scale research is needed. The policy makers should integrate mental health programs into routine HIV care for early identification and treatment of depression. The health workers should focus on patients both clinical and social aspect taking presence of social support, social stigma, adverse drug reaction, presence of opportunistic infection and other chronic diseases into great consideration.

## Limitation of the study

Causality cannot be confirmed since the research design is cross-sectional. This study was conducted at single hospital. Thus, the finding cannot be generalized to Ethiopian people and caution is warranted in generalizing the findings to other areas.

## Data Availability

The data used during this study are available on request.

## Abbreviations

AIDS: acquired immunodeficiency syndrome
AOR: adjusted odd ratio
ART: antiretroviral therapy
CI: confidence interval
EFAHAPCO: Federal HIV/AIDS Prevention and Control Office
HIV: human immunodeficiency virus
OI: opportunistic infections
PHQ: Patient Health Questionnaire
PLWHIV: people living with human immunodeficiency virus
SPSS: Statistical package for social science
